# Molecular and biophysical requirements for B cell receptor-dependent enhancement of dengue virus infection

**DOI:** 10.1128/jvi.02216-25

**Published:** 2026-07-20

**Authors:** Gaby Madrigal, Chad Gebo, Benjamin D. McElvany, Menelaos Symeonides, Sean A. Diehl, Adam T. Waickman

**Affiliations:** 1 Department of Microbiology and Molecular Genetics, University of Vermont Larner College of Medicine 169979, Burlington, Vermont, USA; 2 Cellular and Molecular Biomedical Sciences Graduate Program, University of Vermont 2092, Burlington, Vermont, USA; 3 Department of Microbiology and Immunology, State University of New York Upstate Medical University 827267, Syracuse, New York, USA; 4 Vaccine Testing Center, University of Vermont Larner College of Medicine 12352 , Burlington, Vermont, USA; 5 Translational Global Infectious Diseases Research Center, University of Vermont 2092, Burlington, Vermont, USA; 6 Global Health Institute, State University of New York Upstate Medical University 12302, Syracuse, New York, USA; Wake Forest University School of Medicine, Winston-Salem, North Carolina, USA

**Keywords:** BCR-dependent enhancement, BCR, B cell, immune-mediated enhancement, DENV, dengue virus

## Abstract

Dengue virus (DENV) is the causative agent of dengue, a mosquito-borne disease that represents a significant and growing public health burden around the world. A unique pathophysiological feature of dengue is immune-mediated enhancement, wherein preexisting immunity elicited by a primary infection can enhance the severity of a subsequent infection by a heterologous DENV serotype. A leading mechanistic explanation for this phenomenon is antibody-dependent enhancement (ADE), where sub-neutralizing concentrations of DENV-specific IgG antibodies facilitate the entry of DENV into FcγR-expressing cells. Accordingly, this model posits that phagocytic mononuclear cells are the primary reservoir of DENV. However, multiple independent groups have shown that B cells are the largest reservoir of virally infected cells in circulation during acute dengue, representing a disconnect in our understanding of immune-mediated DENV tropism. In response to this persistent knowledge gap, our team has previously identified a novel mechanism of immune-mediated enhancement we have termed B cell receptor (BCR)-dependent enhancement (BDE) of DENV infection. In this study, we show that DENV infection of DENV-reactive B cells is highly sensitive to BCR/DENV envelope (E) protein interactions. DENV entry into this subset of B cells is dynamin-mediated, requires proximal BCR signaling, and involves localization to late endosomes. Finally, we show that immortalized DENV-reactive B cells are productively infected by live DENV, capable of supporting active viral replication of all four serotypes. We propose that BDE provides an additional layer of pathogen-specific immune-mediated infection risk that complements existing models of ADE and offers additional insight into potential mechanisms of DENV immunopathogenesis.

**IMPORTANCE:**

This study provides mechanistic insight into the process of B cell receptor (BCR)-dependent enhancement of dengue virus (DENV) infection. Immune-mediated enhancement of infection is a cornerstone of dengue immunopathogenesis and is most often attributed to antibody-dependent enhancement (ADE). BCR-dependent enhancement of DENV infection is an alternative and complementary mechanism for immune-mediated enhancement that addresses several persistent knowledge gaps in the dengue field and may offer mechanistic insight into why some people progress to severe dengue after infection.

## INTRODUCTION

Dengue virus (DENV) is a prevalent arboviral pathogen that represents a significant public health burden in many regions of the world. An estimated 3.6 billion people live in regions with sustained DENV transmission, with the virus infecting an estimated 400 million individuals every year (1, 2). Transmitted via the bite of infected *Aedes* mosquitoes, DENV co-circulates as four genetically and immunologically distinct serotypes: DENV-1, -2, -3, and -4. Approximately 25% of DENV infections are thought to result in some form of symptomatic illness, ranging in severity from dengue fever—a mild flu-like presentation of infection—to life-threatening severe dengue, dengue hemorrhagic fever, or dengue shock syndrome (DSS) (3–5). Currently, there are no accurate prognostic indicators of which patients will progress to develop severe dengue. However, the risk of developing severe dengue increases in patients previously infected by a different (heterologous) serotype of dengue compared to DENV-naive individuals (1, 6). This increased risk of severe disease following heterologous reinfection strongly suggests a mechanism of immune-mediated enhancement of DENV infection.

While the etiology of severe dengue is complex and incompletely understood, *in vitro* studies and epidemiological modeling suggest that antibody-dependent enhancement (ADE) of DENV infection may play a significant role in both the risk of DENV infection and in dengue pathogenesis (6–9). Waning titers of cross-reactive DENV-specific antibodies to sub-neutralizing levels have been posited to result in opsonization of the virion and subsequent uptake by Fcγ-receptor (FcγR)-bearing cells such as monocytes, macrophages, and dendritic cells (10, 11). This has been suggested to result in both an increase in the number of infected cells early after inoculation and a qualitatively altered intrinsic response to infection in these susceptible phagocytes (12–14). The delicate balance between protective and enhancing levels of immunity in dengue is a hallmark of the disease and has complicated dengue control efforts for decades.

While the ADE model of dengue immunopathogenesis has been a cornerstone of the field for nearly 50 years, numerous observations strongly suggest that other immunologic mechanisms must also contribute to DENV pathology. Most notably, while *in vitro* infection studies and histological analysis of samples collected from individuals who have succumbed to severe dengue have shown a high DENV burden in FcγR-expressing phagocytes (12, 15–18), *ex vivo* analysis of PBMC collected during acute DENV infection instead shows that B cells are the dominant circulating cellular reservoir of DENV (12, 19–23). This observation has been recapitulated by multiple groups—including our own—using an array of assays including flow cytometry, scRNA-seq, intrathoracic inoculation of mosquitoes with purified cell populations, and qRT-PCR (12, 19–23). While B cells express an FcγR, the specific isoform of this receptor (FcγRIIb) has been shown to be incapable of facilitating ADE and instead results in anergy and apoptosis in B cells when engaged (20, 24). Accordingly, there is a need for the field to reconcile these disparate observations and assumptions regarding DENV cellular tropism (12, 20, 23).

Recently, our team identified a novel DENV entry mechanism specifically within DENV-reactive B cells, a process we have termed B cell receptor-dependent enhancement (BDE). Our group, as well as others, have shown that viruses are able to utilize B cell receptor (BCR) antigen specificity in order to preferentially infect subsets of the B cell populations in a highly antigen-specific fashion (25, 26). We have shown *in vitro* that B cells expressing a DENV envelope protein-specific BCR were susceptible to DENV infection and that the frequency of DENV-infectible B cells in PBMCs increased significantly after a primary DENV infection (25). However, questions remain regarding the signaling pathways and cellular processes required for BCR-mediated infection of DENV-specific B cells.

In this study, we define the cell-intrinsic mechanistic requirements for BCR-mediated entry of DENV into DENV-specific B cells utilizing a unique set of immortalized flavivirus-specific human B cells. Using this model system, we confirm that the susceptibility of these cells is dependent both on the antigen specificity of the BCR, as well as canonical BCR signaling and endocytic pathways. We show that infection of B cells leads to the localization of DENV into late endosomes, an essential part of the DENV entry cycle. Finally, we demonstrate that infected B cells generate infectious virus particles for all DENV serotypes. Overall, our results highlight the entry and signaling requirements in BDE and emphasize how generation of DENV-specific immunologic memory may play a critical cell-intrinsic role in determining DENV susceptibility at an organismal level.

## RESULTS

### BCR antigen specificity determines DENV susceptibility of B cells

In our prior work, we demonstrated that ectopic expression of DENV-specific BCRs rendered normally non-infectible cells highly susceptible to DENV infection (25). Furthermore, we determined that immortalized human B cell lines naturally expressing DENV-specific BCRs were uniquely and potently susceptible to DENV infection (25). To further define the mechanistic requirements for this BCR-dependent infection process, we further examined these flavivirus-reactive immortalized B cells, generated from individuals previously infected with DENV-1 and DENV-2 (7B9^DENV^ cells) or Zika virus (ZIKV, 2F3^ZIKV^ cells).

**Fig 1:**
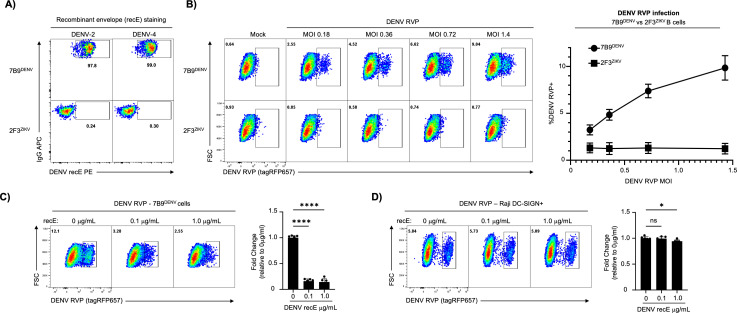
BCR-dependent enhancement in DENV-reactive B cells is highly sensitive to DENV envelope-specific interactions. (**A**) Binding of conformational recombinant E dimers (recE) from DENV-2 (left) and DENV-4 (right) to 7B9^DENV^ and 2F3^ZIKV^ immortalized B cells. (**B**) Flow cytometric analysis of DENV-4 pseudotyped reporter virus particle (RVP) titration on 7B9^DENV^ and 2F3^ZIKV^ cells. Data collected 24 h post-infection. (**C**) Flow cytometric analysis of DENV-4 RVP-infected 7B9^DENV^ cells pre-treated with 0.1 μg/mL or 1.0 μg/mL of DENV-2 recE before the addition of DENV-4 RVP. (**D**) Flow cytometric analysis of Raji DC-SIGN + cells pre-treated with 0.1 μg/mL or 1.0 μg/mL of DENV-2 recE before the addition of DENV-4 RVP. Error bars ± SEM. ns = not significant, *P < 0.05, ****P < 0.0001, One-way ANOVA with Dunnett’s multiple comparisons test, with a single pooled variance. Experiments were conducted across two biological replicates in duplicate and/or triplicate.

Consistent with the binding specificity of the secreted antibody produced by these cells (25), we observed robust binding of recombinant DENV-2- and DENV-4-stabilized E dimer proteins (recE) (27) to the surface of the 7B9^DENV^ cells, but not 2F3^ZIKV^ cells (Fig. 1A; Fig. S1). Mechanistically, if this epitope/paratope interaction contributed to the process of BDE, we hypothesized that blocking DENV-E protein binding sites on the BCR of DENV-specific B cells would result in a reduction in the susceptibility of DENV-reactive B cells to DENV infection. To facilitate the specific quantification of productive infection—rather than antigen-binding/uptake—we utilized a DENV-pseudotyped reporter virus particle (RVP) platform. This system encodes a fluorescent reporter expressed only upon successful cytoplasmic entry and translation, allowing specific quantification of productive infection rather than surface binding alone (28, 29). Consistent with our previously published results (25), the 7B9^DENV^ cells were highly susceptible to DENV RVP infection, while the 2F3^ZIKV^ cells exhibited no RVP infection across all tested infection conditions (Fig. 1B; Fig. S2).

We next incubated 7B9^DENV^ cells with recE prior to the addition of DENV RVP. As hypothesized, we observed a dose-dependent reduction in the frequency of RVP-infected cells in the presence of recE blockade (Fig. 1C). To confirm that this reduction in infection was due to the BCR specificity of the 7B9^DENV^ cells and not due to other antagonistic interactions, we repeated this recE blockade in a known DENV-susceptible Raji DC-SIGN+ cell line. DC-SIGN/DC-SIGNR-mediated DENV uptake, unlike BCR-mediated uptake, depends on low-affinity, high-valency interactions with E protein carbohydrates rather than specific epitope recognition (30, 31). Accordingly, we predicted—in contrast to what was observed for BCR-mediated infection—that DC-SIGN/DC-SIGNR-mediated infection would be minimally impacted by the exogenous addition of soluble recE. Consistent with this model, we only observed a modest reduction in RVP infection of DC-SIGNR-expressing Raji cells (Fig. 1D), emphasizing the specificity and potency of BCR-mediated DENV uptake and subsequent infection.

### BCR signaling is required for BDE-mediated viral entry

Having demonstrated the role of BCR specificity in DENV infection of immortalized human B cells, we next sought to define the cell-intrinsic signaling and biophysical processes required for BDE. We posited that BCR-mediated infection of DENV-specific B cells would be dependent on canonical B cell signaling and endocytic processes including dynamin-mediated endocytosis and Bruton’s tyrosine kinase (BTK) signaling. However, we predicted that other downstream BCR signaling processes not directly involved in endocytosis—such as PI3K signaling—would be dispensable for the initial entry of DENV into the B cell, although these pathways may be involved in regulating the permissivity of the cell to DENV infection due to their role in regulating protein translation and cell cycle progression.

**Fig 2:**
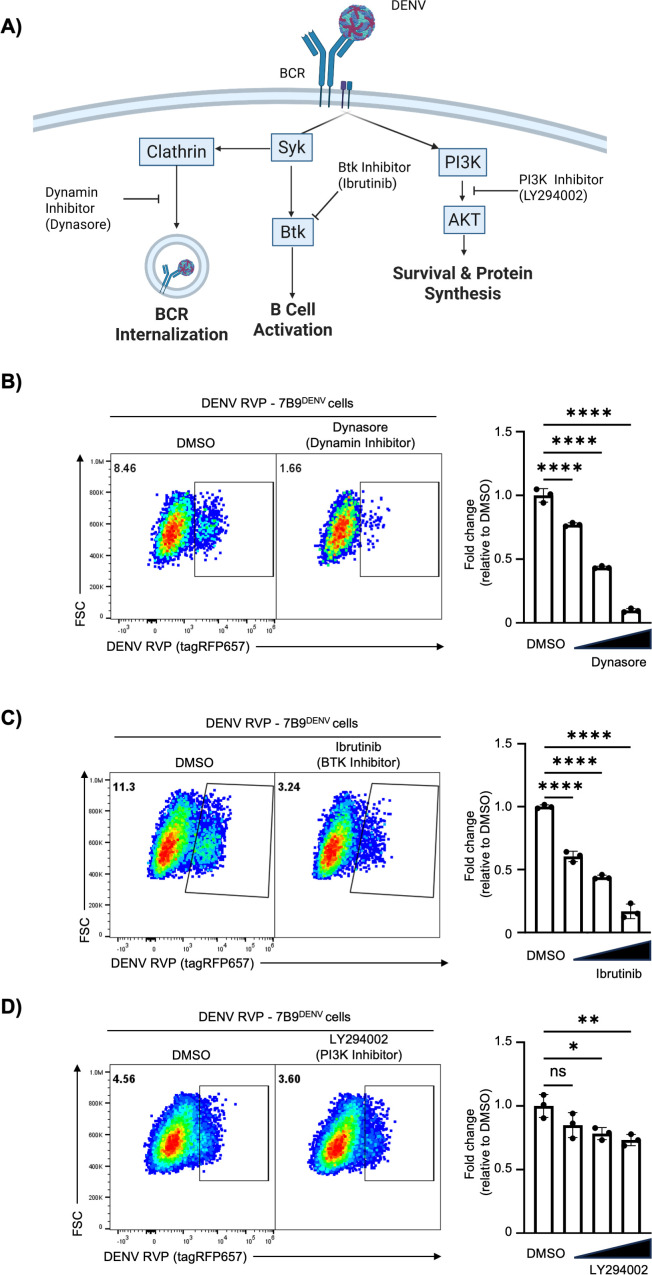
Proximal BCR signaling required for viral entry in DENV-reactive B cells. (**A**) Schematic representation of BCR signaling pathways and targets of pharmacological inhibitors. Cells were pre-treated with the selected inhibitor for 30 min prior to the addition of DENV-4 RVP. (**B**) Flow cytometric analysis of DENV-4 RVP infection of 7B9^DENV^ B cells pre-treated with 12.5, 50, or 200 µM of dynamin inhibitor Dynasore. (**C**) Representative flow plots of DENV-4 RVP infection of 7B9^DENV^ B cells pre-treated with 1.56, 12.5, or 50 µM of the BTK inhibitor ibrutinib. (**D**) Flow cytometric analysis of DENV-4 RVP infection of 7B9^DENV^ B cells pre-treated with 1.56, 12.5, or 50 µM of the PI3K inhibitor LY294002. Error bars ± SEM. ns = not significant, *P < 0.05, *P < 0.01, ****P < 0.0001, One-way ANOVA with Dunnett’s multiple comparisons test, with a single pooled variance. The experimental data shown are representative of two biological replicates performed in triplicate.

To address these questions, we utilized pharmacological inhibitors of endocytosis and proximal BCR signaling, specifically targeting dynamin-mediated endocytosis, Bruton’s tyrosine kinase (BTK) signaling, and phosphoinositide 3-kinase (PI3K) signaling (Fig. 2A). 7B9^DENV^ cells were pre-treated for 30 min with the indicated inhibitors prior to the addition of the DENV RVP, followed by an additional 4 h of incubation in the presence of inhibitors for infection. Following extensive washing and trypsin treatment to remove any remaining surface-bound RVP, the cells were incubated for an additional 20 h and analyzed by flow cytometry to quantify the frequency of DENV RVP-infected cells.

Consistent with our hypothesized mechanism of action for BDE, inhibition of dynamin-mediated endocytosis with the drug Dynasore resulted in a significant and dose-dependent reduction in DENV RVP-infected 7B9^DENV^ cells (Fig. 2B). This observation is consistent with the endocytic pathways utilized by DENV in other cell types (32, 33). Next, we sought to assess the contribution of proximal BCR-specific signaling on BDE and observed a similar reduction in the frequency of RVP-infected cells upon inhibition of BTK signaling with ibrutinib (Fig. 2C). However, only a modest reduction in RVP infection was observed in cells treated with the PI3K inhibitor LY294002 (Fig. 2D), despite a significant reduction in downstream ribosomal S6 phosphorylation (Fig. S3A through C). This suggests that while PI3K activity can be induced following BCR ligation by DENV, it is mostly dispensable for the initial infection of B cells via BDE. Notably, cell viability was not significantly impacted in the treated cells, except for the highest concentration of Dynasore (Fig. S3D). To confirm that the reduction in DENV RVP signal observed upon drug treatment was not due to an overall decrease in protein translation or altered cellular homeostasis, we also quantified the expression of green fluorescent protein (GFP) within the 7B9^DENV^ cells, which is driven by a constitutive promoter contained within the retrovirus utilized to immortalize the cells. GFP expression remained stable across all conditions, indicating that the observed reductions in RVP signal resulted from decreased viral entry rather than global disruption of protein translation or cellular homeostasis (Fig. S3E).

### Endosomal localization of DENV in B cells

**Fig 3:**
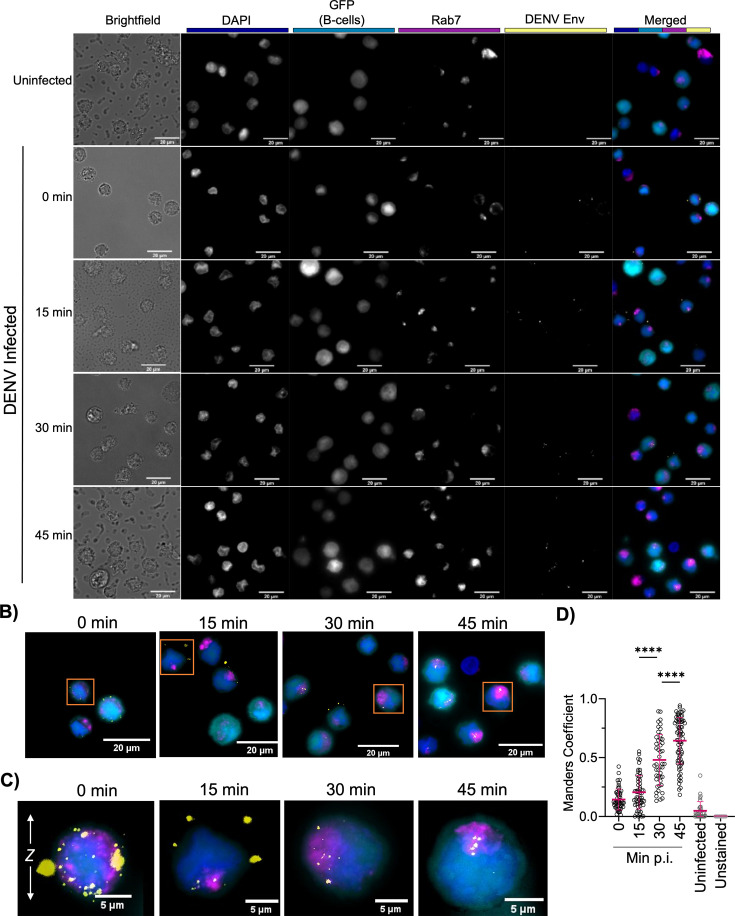
Late endosomal localization of DENV following infection of B cells. 7B9^DENV^ cells were infected with DENV-2 NGC at MOI = 10 at 4°C for 1 h, followed by temperature shift to 37°C to promote viral internalization. At the indicated times, cells were stained for DENV Env, Rab7, and DAPI. GFP is expressed by 7B9 cells. (**A**) Representative fields are shown: brightfield (grayscale), DAPI (blue), GFP+ B cells, Rab7 (red), and DENV Env (yellow), and a merge of fluorescent channels. Scale bars: 20 μm. (**B**) Representative merged channel widefield single z-stack images used for colocalization analysis for DENV Env and Rab7 in GFP + B cells. Scale bar, 20 μm. Cells in orange frames were selected for 3D renders. (**C**) Maximum 3D projections rendered from 38 z-stacks of DENV Env spatial localization over time, beginning outside of cells and ending in the late endosome by the end of the experiment. Scale bars: 5 μm. (**D**) Manders correlation analysis of DENV Env overlapping with Rab7 from approximately 50 frames per time point, with ~60 B-cells per frame. Lines = mean; error bars = standard deviation of the mean. Uninfected and unstained groups used for thresholding were pooled from all time points. Data were analyzed by one-way ANOVA with Kruskal-Wallis correction for multiple comparisons. ****P < 0.0001.

Having demonstrated the importance of BCR specificity and proximal BCR signaling to the process of BDE, we investigated the timing and subcellular trafficking of DENV following BCR engagement. We hypothesized that DENV/BCR engagement would lead to receptor internalization and subsequent localization to the late endosome, a necessary part of the DENV entry lifecycle (34). To test this, 7B9^DENV^ cells were incubated with DENV-2 at 4°C for 1 h to allow for surface virus binding. After washing and temperature shift to 37°C, we analyzed DENV localization to late endosomes by staining for anti-DENV E protein and Rab7, a well-established marker of the late endosome (34, 35), by immunofluorescence microscopy (Fig. 3A). DENV was detected extracellularly at 0–15 min, with intracellular accumulation of DENV observed at 15–30 min, colocalization of DENV and Rab7 becoming apparent at 30 min with a high degree of colocalization of DENV and Rab7 observed 45 min post-temperature shift (Fig. 3B). Three-dimensional rendering across z-stacks confirmed intracellular colocalization of DENV and Rab7 in endosomes at 30 and 45 min post-internalization (Fig. 3C; Video S1). Manders correlation coefficient analysis for Rab7 and DENV showed a significant increase in DENV-2 and Rab7 colocalization beginning at 30 min post-infection in DENV-specific 7B9^DENV^ human B cells, with the strongest correlation observed 45 min post-temperature shift (Fig. 3D). These results show that over time, DENV-2 bound to antigen-specific BCRs becomes internalized and localizes to late endosomes.

### B cells are productively infected with DENV via BDE

In our previous work, we demonstrated the presence of positive- and negative-strand DENV RNA in immortalized B cells expressing a DENV-reactive BCR that were exposed to DENV-2 (25). In contrast, no DENV RNA was detected in B cells expressing a ZIKV-reactive BCR following incubation with DENV-2. These results suggest that immortalized DENV-specific B cells may be capable of supporting DENV replication in a BCR-dependent manner. Additionally, since BCR signaling was involved in early infection steps and DENV was internalized in 7B9^DENV^ cells, as mentioned above, we hypothesized that these cells may complete DENV replication, resulting in infectious virion production. To test this, we exposed both 7B9^DENV^ cells and 2F3^ZIKV^ cells to DENV-2 at a high MOI for 24 h, consistent with the conditions utilized in our previously published scRNA-seq analysis (25). Irradiated CD40L-expressing L cell fibroblasts used as feeder cells for B-cell maintenance were exposed in a similar fashion as a negative infection control, while Vero cells were utilized as a positive control. Following DENV exposure and washing, supernatants from cells at different times after infection were inoculated onto Vero cells to determine the presence of infectious DENV by a focus-forming unit assay (FFU). We observed robust DENV-2 production from the 7B9^DENV^ cells starting 24 h after initial inoculation, similar in magnitude to what was observed from inoculated Vero cell cultures (Fig. S4). Although lower than 7B9 cells, we observed infectious DENV-2 in 2F3^ZIKV^ and CD40L feeder cell cultures, indicating residual inoculum from the high MOI used in this experiment.

**Fig 4:**
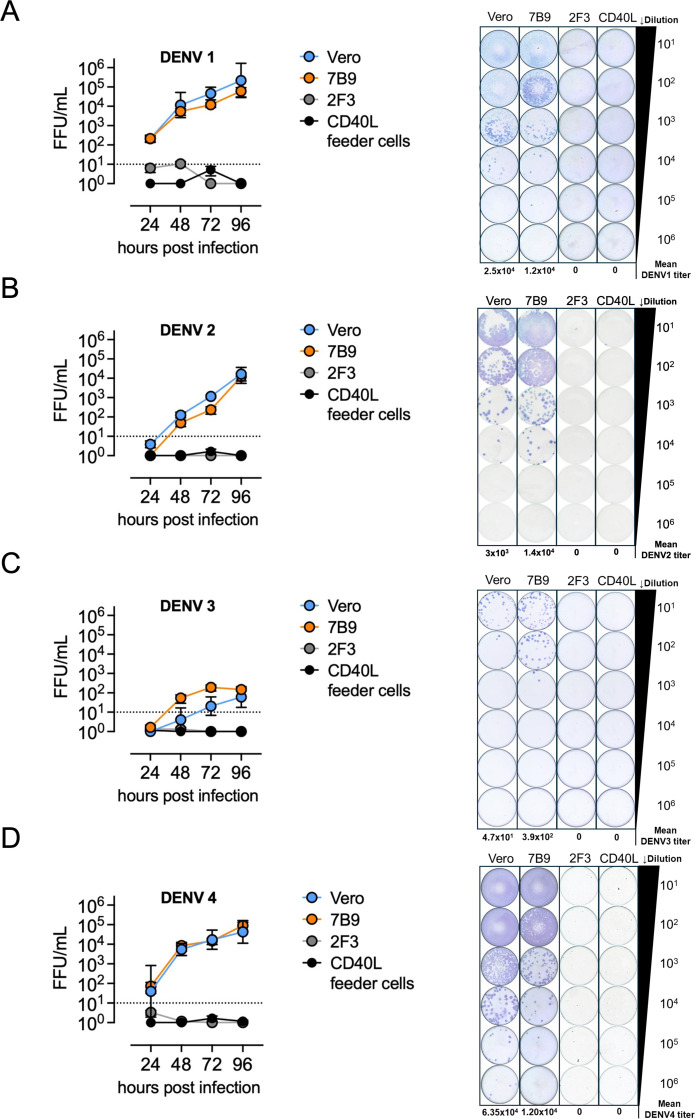
Generation of infectious DENV-1 to 4 by infected DENV-specific B cells. Vero cells, 7B9^DENV^ B cells, 2F3^ZIKV^ B cells, or CD40L-expressing fibroblast feeder cells were inoculated with (**A**) DENV-1/Western Pacific, (**B**) DENV-2/NGC, (**C**) DENV-3/Sleman, or (**D**) DENV-4/Dominica81, at MOI = 0.1 for 1 h at 37°C, washed, and cultured for up to 4 days. At the indicated times, infectious virus titers in B cell supernatants were determined by passage on Vero cells and assessed using a standard focus assay with data presented as FFU/mL. Representative focus assays from 72 h post-infection supernatants are depicted to the right of the graphs. Experiments were performed in triplicate, each with two technical replicates. Mean ± standard deviation is shown. Dashed lines in graphs indicate the FFU assay limit of detection.

We next employed more stringent infection conditions to avoid potential cytotoxicity or cell stress associated with high viral levels and to reduce residual carry-through of viral inoculum. Also, since the 7B9^DENV^ BCR binds to all four serotypes of DENV (25), we next assessed the capacity of 7B9^DENV^ cells to support replication of other serotypes. Cells were incubated with DENV-1, -2, -3, or -4 at an MOI of 0.1 for 1 h, followed by extensive washing and additional incubation of infected cultures for up to 4 days. Supernatants were tested as above for the presence of infectious virus by focus-forming assay by inoculation on Vero cells. Under these more stringent conditions, we observed no carry-through viral inoculum (CD40L-feeder cell condition) or production of infectious DENV-1 to -4 by 2F3^ZIKV^ cells (Fig. 4A through D). In contrast, 7B9^DENV^ cells exhibited sustained production of infectious DENV-1 to 4 at levels similar to those observed for Vero cells inoculated under the same conditions (Fig. 4A through D). These results demonstrate that DENV-specific B cells can support the replication of all DENV serotypes for at least 96 h *in vitro*.

## DISCUSSION

In this study, we provide direct evidence that dengue virus (DENV) can productively infect human B cells in a fashion dependent on BCR specificity and proximal BCR signaling. These findings expand the current paradigm of dengue immunopathogenesis, which has historically emphasized ADE in FcγR-bearing myeloid cells, by providing mechanistic insight into how B cells themselves serve as a cellular reservoir for DENV replication. Previous studies have reported non-neutralizing DENV-specific antibodies facilitating ADE, but the role of B cells as direct targets of infection has remained underappreciated. By demonstrating productive replication and infectious particle release from DENV-specific B cells, our work provides mechanistic support for clinical observations of B cell tropism.

These results support a growing body of literature emphasizing B cells as the principal circulating cellular reservoir of DENV-infected cells (12, 19–23). Our findings work in conjunction with existing models of ADE, expanding on our understanding of DENV cellular tropism and explaining why we see such a large prevalence of DENV-infected but non-ADE-susceptible cells in circulation during acute illness. We posit that these highly susceptible B cells generated after the initial exposure aid in the establishment of DENV infection during secondary infection, where the frequency of these highly susceptible cells, paired with other risk factors such as waning neutralizing antibody titers, could synergize to increase the risk of severe disease.

While this study provides additional mechanistic insight into BDE, several facets of the phenomenon require further characterization. Although we observed that proximal BCR signaling is required to establish infection, it remains unclear whether BCR signaling also influences the intrinsic permissivity of DENV-specific B cells to DENV infection. An analogous process, referred to as “intrinsic” ADE, has been described in which differential signaling through the FcR induces metabolic changes that create a more favorable environment for DENV replication. Understanding how BCR engagement impacts this process will be important, particularly given our observation that BTK inhibition decreases viral entry. Although we observed that PI3K inhibition did not affect entry, DENV can induce or dysregulate PI3K activation in other cell types (36). Given PI3K’s critical role in regulating cell survival, cellular metabolism, and protein translation, this pathway likely influences DENV replication and maturation within BDE-infected cells.

Emerging evidence suggests that DENV infection may impact B cell function, but the mechanisms and consequences of these changes remain incompletely understood. DENV-infected B cells from patients with severe disease exhibited higher expression of genes encoding activation markers, including CXCR4, CD69, FCRL1, and IRF1 (21). DENV antigen-positive B cells from dengue patients showed increased *ex vivo* markers of proliferation and enhanced differentiation into plasmablast/plasma cells after culture *in vitro* (20). Finally, B cells of children with severe disease showed a paradoxical upregulation of interferon response genes as well as those involved in host and viral translation and RNA binding, whereas many human leukocyte antigen genes were downregulated, suggesting impaired antigen presentation (37). We also previously observed negative Z-scores for pathways involved in phagocytosis and BCR signaling components in 7B9^DENV^ cells infected with DENV at a high MOI (25). Taken together, we postulate that DENV infection may impact multiple aspects of B-cell function, including activation and differentiation—areas that require further investigation. Beyond these intrinsic effects, it will also be important to understand how DENV infection via BDE impacts immunologic memory, particularly given the shift from poorly neutralizing, type-specific responses to broadly neutralizing, cross-reactive responses observed following secondary infection (38).

A striking finding in this study is that DENV-specific 7B9 B cells produced infectious virus at levels comparable to interferon-deficient Vero cells when inoculated at identical MOIs. We propose that this similar infectious output arises from complementary but distinct cellular properties. The 7B9 cells exhibit markedly elevated susceptibility to DENV infection compared to Vero cells, resulting from BCR-mediated viral entry—a high-affinity, antigen-specific mechanism absent in Vero cells. Conversely, Vero cells likely demonstrate superior permissivity to DENV replication due to their well-documented deficiency in type I interferon (IFN) signaling, which normally restricts viral replication. Thus, the apparent equivalence in infectious titers between these cell lines may reflect an interesting trade-off: 7B9 cells achieve robust infection through enhanced viral entry (susceptibility), while Vero cells achieve similar replication through attenuated antiviral defenses (permissivity). These complementary mechanisms highlight distinct pathways through which cells can support high-level DENV replication, each relevant to understanding DENV tropism in different cellular contexts during infection.

While this study focused on the molecular and biophysical requirements for BDE, it is probable that these same mechanisms and signaling pathways may fundamentally alter DENV pathogenesis in a cell-intrinsic fashion. While not directly assessed in this study, BCR ligation via BDE may trigger metabolic reprogramming in DENV-specific B cells, similar to that observed during canonical B cell activation (39, 40). Furthermore, BCR crosslinking promotes upregulation of many biosynthetic pathways, particularly protein synthesis machinery, which normally prepare differentiating B cells for antibody secretion upon differentiation into plasmablasts/plasma cells (39, 40). However, when a DENV-specific B cell is infected via BDE, this BCR-driven metabolic shift may be co-opted to support viral replication and production of infectious progeny virions. Additionally, BCR signaling results in the dramatic upregulation of cellular programs such as the unfolded protein response (UPR) (41), potentially allowing sustained DENV replication and increased secretion of both infectious particles and viral accessory proteins (e.g., NS1). These altered cellular conditions may thus represent a permissive environment that enhances viral burden and potentially contributes to dengue pathogenesis.

There are some limitations to this study that highlight the need for additional analysis. Namely, the DENV-specific human B cells utilized for these analyses were immortalized by retroviral transduction. These cells were indispensable for the analysis described in this manuscript due to the specificity of their BCR, which allowed for minimal manipulation, as well as their functional BCR signaling (25, 42). However, these immortalized IgG memory cells are maintained in a partial plasmablast-like state through persistent culturing alongside CD40L-expressing cells and IL-21 (25, 42). These *in vitro* cultures do not recapitulate the heterogeneity of primary human B cells, requiring further characterization in more biologically relevant models. Additionally, BCR engagement and internalization activate a complex signaling network; further study of other BCR signaling components—such as Lyn, Syk, SHP1, and Ca²^+^ flux—is required to understand the full impact of BCR signaling on DENV entry. Finally, the downstream consequences of B cell infection by DENV, including the effects on antibody production, apoptosis, and immune regulation, remain to be elucidated.

In conclusion, this study has further characterized the phenomenon of BCR-dependent enhancement as a novel mechanism of viral entry for DENV. This mechanism expands our understanding of how the role of immunologic memory shapes DENV susceptibility and how the presence of DENV-specific memory B cells may influence an individual’s risk of infection or progression to severe disease. This study highlights the need to understand not only the contribution of antibodies as mediators of protection and pathogenesis in dengue but also how B cells themselves may contribute to risk and protection from DENV infection.

## MATERIALS AND METHODS

### Viruses and RVPs

DENV-1/WestPac (GenBank AY145121.1), DENV-2/NGC, DENV-3/Sleman/78 (AY648961.1), and DENV-4/Dominica81 (AF326573.1) stocks were prepared by propagating a low-passage inoculum (MOI = 0.5) in Vero cells maintained in OPTI-PRO serum-free medium (Invitrogen, cat. no. 12309019) supplemented with 2% fetal bovine serum (Atlanta Biologicals/R&D Systems, cat. no. S11150) and GlutaMAX (Gibco, cat. no. 35050061) at 37°C, 5% CO_2_ in a humidified incubator. Viruses were titrated by FFU assay as described (43). DENV-4 (strain Dominica) tagRFP657 RVP was prepared as previously described (28). RVPs were titrated on Raji DC-SIGN+ cells, infectious units (IU) calculated based on the dilution of virus needed to infect 15%–20% of cells as previously described (44).

### Cell lines

Raji cells overexpressing DC-SIGN+ (CD209L) (BEI, ARP-9945) were cultured in complete RPMI-1640 medium (complete RPMI) supplemented with 10% FBS and 100 U/mL penicillin-streptomycin. All cells were cultured at 37°C, 5% CO_2_, and 85% humidity. 7B9^DENV^ is an immortalized human monoclonal memory B-cell line expressing an IgG isotype BCR that binds, but poorly neutralizes, DENV-1 to -4 and ZIKV generated from a flavivirus-naïve volunteer who was vaccinated with DENV-1Δ30 and challenged 270 days later with DENV-2Δ30 Tonga (NCT02392325). B cells were isolated 90 days following DENV-2Δ30 challenge (25). 2F3^ZIKV^ is an immortalized human monoclonal memory B-cell line expressing a ZIKV-specific IgG isotype BCR that binds ZIKV, but not DENV, and was obtained from an exposed volunteer (DT165) approximately 1 year following ZIKV infection (25). Immortalized memory B cells were previously generated by transduction of CD27+IgM– memory B cells with a retrovirus encoding human B cell lymphoma (BCL)-6 and BCL-xL and enhanced GFP (42). B cells were maintained in Iscove’s-modified Dulbecco’s medium (IMDM, Invitrogen, cat. no. 12200-028) supplemented with 8% FBS and 1% pen/strep in 24-well plates with 100,000 irradiated CD40L-expressing mouse L cell fibroblasts and 25 ng/mL rhIL-21 (Peprotech, cat. no. 200-21-1MG) as previously described (42).

### Flow cytometry

Immortalized B cells were stained for viability (L/D) with Fixable Viability Dye eFluor 780 (Invitrogen, cat. no. 65-0865-14) according to the manufacturer’s protocol and washed with FACS buffer (PBS + 1% BSA); 100 ng of AviTagged and biotinylated DENV-2 or DENV-4 recombinant E protein stabilized dimers (DV2 SC.14 and DV4 SC, respectively, a kind gift of Brian Kuhlman and Aravinda DeSilva, University of North Carolina, Chapel Hill (27)) were labeled with 0.5 μL of streptavidin-PE (Invitrogen, cat. no. 12-4317-87) in a volume of ~8 µL for 30 min on ice. 100,000 B cells were then stained with PE-labeled recE on ice for 30 min. Following a wash with FACS buffer and final staining with anti-human IgG-APC (clone: M1310G05, BioLegend, cat. no. 410712) for 30 min. Events were captured on a Beckman Cytoflex at the Microscopy Imaging and Cytometry at the University of Vermont (RRID# SCR_018821) and analyzed with FlowJo version 10.

DENV-4 RVP infection assays were collected on a Thermo Fisher Attune NXT flow cytometer, detecting 7B9^DENV^ or 2F3^ZIKV^ cells through detection of GFP. RVP infection was detected through the expression of tagRFP657. Data collection was performed on a Thermo Fisher Attune NXT flow cytometer and analyzed using FlowJo v10.2 (Becton Dickinson).

### DENV-4 tagRFP657 RVP infection of human B cells

7B9^DENV^ or 2F3^ZIKV^ cells were plated at 5 × 10^4^ cells per well. DENV-4 RVP was titrated in 2-fold serial dilutions. Cells were incubated for 4 h at 37°C, followed by washing and trypsinization to remove remaining surface-bound RVP. The cells were then incubated for an additional 20 h at 37°C. Cells were washed twice with PBS + 2% FBS, then fixed using 4% PFA and detected by flow cytometry. MOI calculation for titration was back-calculated based on IU calculations on Raji DC-SIGNR cells.

### DENV-2 recombinant E dimer infection blockade

DENV-2 recombinant envelope protein dimers (recED) were received under MTA from Aravinda de Silva at the University of North Carolina, Chapel Hill. 7B9^DENV^ or Raji DC-SIGN+ cells were plated at 5 × 10^4^ cells per well and chilled to 4°C before no treatment or addition of 0.1 μg/mL or 1.0 μg/mL of the DENV-2 recED. Cells were incubated at 4°C for 30 min, followed by the addition of DENV-4 RVP at an MOI of 10. Cells were incubated for an additional 30 min at 4°C before three washes using the cell culture medium. Cells were resuspended in fresh cell culture media and incubated for 24 h at 37°C. Cells were washed twice with PBS + 2% FBS before fixation using 4% PFA and analyzed by flow cytometry.

### Inhibition of viral entry by pharmacological inhibitor treatment

7B9^DENV^ cells were plated at 5 × 10^4^ cells per well and pre-treated for 30 min at 37°C with DMSO or various concentrations of pharmacological inhibitors: 12.5, 50, and 200 µM of dynamin inhibitor Dynasore (Abcam, cat. no. ab120192); 1.56, 12.5, and 50 µM of BTK inhibitor Ibrutinib (Selleck, cat. no. S2680); or 1.56, 12.5, and 50 µM of PI3K inhibitor LY294002 (Selleck, cat. no. S1105). Following pre-treatment, DENV-4 RVP was added at an MOI of 2.5 and incubated for 4 h in the presence of the drug. Cells were then washed twice using cell culture medium and trypsinized and then washed again to remove surface-bound RVP before resuspension in fresh cell culture medium and incubated at 37°C for 20 h. Cells were then washed twice with PBS + 2% FBS before fixation using 4% PFA and analyzed by flow cytometry.

Inhibitor treatment effect on cell viability in the absence of RVP was performed under the same conditions as described above, then assessed by metabolic conversion of MTT tetrazolium into formazan utilizing the CellTiter 96 Non-Radioactive Cell Proliferation Assay (MTT) (Promega, cat. no. G4000). 1% Triton-X (Thermo Fisher, cat. no. A16046.AE) was used as a positive control for cell death. Data were collected on a Biotek µQuant plate reader at 570 nm.

Pharmacological inhibition of PI3K was assessed via flow cytometry through the detection of phosphorylated ribosomal protein S6 at Ser235 and Ser236 as a direct downstream measurement of activated PI3K. Cells were treated with DMSO (Thermo Fisher, cat. no. 036480.AP) or 10 µM LY294002 for 30 min. They were then washed twice with PBS + 2% FBS. Then fixed using 4% PFA and stained utilizing AF647-labeled phospho-S6 monoclonal cupk43k (Thermo Fisher, cat. no. 740057MP647) diluted 1:1,000 in PBS + 2% BSA. Data collection was performed on a Thermo Fisher Attune NXT flow cytometer and analyzed using FlowJo v10.2 (Becton Dickinson).

### Detection of DENV internalization and endosomal localization by microscopy

Cellvis 8-well chamber slides (Cellvis, cat. no. C8-1.5H-N) were coated with Poly-L-Lysine (Cultrex #3438-100-01) diluted 1:10 in Milli-Q water to adhere cells to the slide. 7B9^DENV^ or 2F3^ZIKV^ cells were chilled to 4°C for 30 min, followed by the addition of DENV-2 NGC at an MOI = 10 and incubation for 1 h at 4°C to allow surface binding. Cells were then washed thrice with cold complete medium and plated at 1 × 10^5^ cells per well and incubated at 37°C for 0, 15, 30, and 45 min to promote virus internalization. After incubation, cells were fixed using 4% PFA in PBS and permeabilized using PBS with 0.1% Triton-X and 1% BSA. Cells were stained overnight at 4°C with primary antibodies diluted in permeabilization (perm) buffer: mouse cross-reactive anti-flavivirus E protein monoclonal antibody 4G2 diluted to 4 µg/mL (generated in-house), and rabbit anti-human Rab7 monoclonal antibody D95F2 diluted 1:200 (Cell Signaling, cat. no. 9091). After primary antibody staining, cells were washed twice in perm buffer and incubated with fluorescently labeled secondary antibodies: donkey anti-mouse IgG AF647 (1:1,000, Jackson ImmunoResearch Inc, cat. no. 715-605-151) and donkey anti-rabbit IgG AF568 (1:1,000, Thermo Fisher, cat. no. A10037). After antibody staining, cells were washed twice in PBS, and nuclei were counterstained with 300 nM DAPI (Thermo Fisher, cat. no. D1306). Cells were then washed twice with PBS. Intracellular GFP cytoplasmic fluorescence was captured to discriminate immortalized B cells (GFP+) from residual irradiated CD40L-expressing mouse fibroblasts (DAPI+GFP−). GFP+ B cells were >85% of total DAPI+ cells on average per analyzed field. Images were collected on a Marianas microscopy system (Intelligent Imaging Innovations, Denver, CO) consisting of a Zeiss Axio Observer 7 stand, motorized XY stage, stage-top incubator chamber (Okolab), X-Cite NOVEM-L LED light source (Excelitas), and Orca Fusion BT sCMOS camera (Hamamatsu). Images were acquired using a 63×/1.4 DIC Plan Apochromat, oil immersion objective, and z-stacks were acquired with a step size of 0.27 µm (37 steps for a total depth of 10 µm). Semrock filter sets were used as follows: DAPI: 392/23 Ex, 433/24 Em; eGFP: 480/17 Ex, 520/35 Em; AF568: 554/23 Ex, 600/37 Em; AF647: 635/18 Ex, and 680/42 Em; all paired with a 409/493/573/652/759-Di01 penta-edge dichroic. Images were rendered using SlideBook 2026.0 (Intelligent Imaging Innovations, Denver, CO) before exporting as raw TIFF images for analysis in Fiji (ImageJ) version 2.16.0 (45, 46).

### Colocalization analysis

Colocalization between Rab7-positive endolysosomal compartments (AF568, Ch4) and DENV virus envelope protein (AF647; Ch5) was analyzed in Fiji (ImageJ) version 2.16.0 (45, 46) with a custom-written macro (File S2) utilizing the JACoP plugin (47) to calculate Manders overlap coefficients (M1 and M2, respectively). M1 represents the percentage of Rab7 (Ch4) signal overlapping with DENV Env (Ch5). M2 represents the percentage of DENV Env (Ch5) signal overlapping with Rab7 (Ch4). Because the biological question centers on the extent of viral internalization and trafficking to Rab7-positive compartments, M2 values were used for all statistical analyses and graphing. To minimize background contributions and ensure accurate overlap quantification, fixed intensity thresholds were applied to each channel prior to coefficient calculation. Thresholds for infected cells were determined empirically from the average of uninfected control images (Ch5 threshold was ≥1,200 mean integrated density), and for stained cells, organelle thresholds were determined from unstained images (Ch4 threshold was ≥10,000 mean integrated density). Thresholds were applied uniformly across the data set; only pixels within these intensity ranges were included in the M1/M2 calculations. M2 values (percentage of DENV Env signal overlapping with Rab7) were exported and analyzed by one-way ANOVA with Kruskal-Wallis correction for multiple comparisons.

### Quantification of infectious DENV

Immortalized B cells (250,000 cells) were cultured in complete IMDM in 24-well plates on a CD40L cell (100,000 cells) monolayer and supplemented with rhIL-21. Irradiated CD40L-expressing L cells (350,000 cells, to account for total cell number in immortalized B cell conditions) cultured in complete IMDM and Vero cells (350,000 cells) cultured in OPTI-MEM 2% FBS and GlutaMAX were included as negative and positive controls, respectively. Cells were exposed to DENV at MOIs of 10 or 0.1 at 37°C for 24 h or 1 h as described in the figure legends. Infected cells were then washed three times with DPBS (Invitrogen, cat. no. 14190-144) and cultured in complete IMDM + rhIL-21 at 37°C for an additional 24, 48, 72, or 96 h. Supernatants were collected at each time point, flash-frozen, and kept at −80°C until further use. Supernatants were diluted in a range of 10-fold dilutions (neat, 10^–1^–10^–7^) in virus titration diluent (Opti-MEM- no GlutaMAX [Invitrogen cat. no. 31985-070], 2% heat inactivated FBS, and 10 mL L-glutamine 200 mM); 100 μL of each dilution was applied to confluent Vero cell monolayers in 24-well plates for 1 h at 37°C, followed by the addition of 1 mL of overlay media (Opti-MEM + GlutaMAX [Gibco, cat. no. 51985.091], 5 g Methylcellulose, 2% heat-inactivated FBS, and 0.5 mL Gentamicin 50 mg/mL [Gibco, cat. no. 15750.060]) and incubation for 4 days at 37°C. Next, the overlay medium was removed by flicking and tapping plates, and monolayers were washed twice with 1× PBS, and fixed with 50%/50% Methanol/Acetone for 40 min at 4°C, followed by blocking for 10 min with antibody dilution buffer (5% Oxoid non-fat dry milk in 1× PBS), washed again twice with PBS, and stained with 4G2 (0.8 ng/mL, HB112 hybridoma from ATCC) in blocking buffer, followed by incubation at 37°C for 1 h. Plates were then washed twice with PBS and incubated with HRP-labeled secondary goat anti-mouse HRP-conjugated antibody (1:2,000, KPL, cat. no. 074-1806) at 37°C for 1 h, washed with PBS, air-dried for 5 min, and treated with 160 µL TrueBlue substrate (SeraCare, cat. no. 5510-0030), allowing foci to develop for ~15 min. Images were captured on a CTL Immunospot S6 (CTL Analyzers, Shaker Heights, OH, USA), and virus titers were calculated as previously described (43).

### Statistical analysis

All statistical analyses were performed using GraphPad Prism Software (GraphPad Software, La Jolla, CA, USA). *P*-value < 0.05 was considered significant. ANOVA was used for the comparison of multiple conditions.

## Data Availability

The authors confirm that the data supporting the findings of this study are available and included within this article.

## SUPPLEMENTAL MATERIAL

File S1 jvi.02216-25-s0001.xlsx Raw data for all figures and plots.

File S2 jvi.02216-25-s0002.docx Fiji macro used for colocalization analysis.

Supplemental figures jvi.02216-25-s0003.pdf Fig. S1 to S4.

Supplemental legends jvi.02216-25-s0004.docx Descriptive legends for Files S1 and S2 and Video S1.

Video S1 jvi.02216-25-s0005.mov Rotating 3D projection of DENV/endosome colocalization.

ASM does not own the copyrights to Supplemental Material that may be linked to, or accessed through, an article. The authors have granted ASM a non-exclusive, world-wide license to publish the Supplemental Material files. Please contact the corresponding author directly for reuse.
