# Supplementary material for: Molecular and biophysical requirements for B cell receptor-dependent enhancement of dengue virus infection
Source: J Virol. 2026 Jul 20;100(8):e02216-25. doi: 10.1128/jvi.02216-25 (PMC13483438; doi:10.1128/jvi.02216-25)
Supplement: Supplementary file 1 [file jvi.02216-25-s0002.docx]

```
Supplemental File 2


// Fiji / ImageJ macro
// Batch JACoP (Manders coefficient)
//
// Channels:
//   ch4 = organelle
//   ch5 = virus
//
// Thresholds:
//   ch4 = 10000
//   ch5 = 1200
//
// Outputs:
//   Image
//   M1_thresh              (JaCoP)
//   M2_thresh              (JaCoP)
//
// CSV saved automatically to input folder

setBatchMode(true);

// ----- settings -----
thra = 10000;
thrb = 1200;

// ----- choose folder -----
inputDir = getDirectory("Choose folder of hyperstacks");
if (inputDir == "") exit("No folder selected.");

fileList = getFileList(inputDir);
if (fileList.length == 0) exit("No files found.");

run("Clear Results");
print("\\Clear");

processed = 0;
skipped = 0;

for (fi = 0; fi < fileList.length; fi++) {

    name = fileList[fi];
    path = inputDir + name;

    if (File.isDirectory(path))
        continue;

    lower = toLowerCase(name);
    if (!(endsWith(lower, ".tif") || endsWith(lower, ".tiff") || endsWith(lower, ".nd2") ||
          endsWith(lower, ".czi") || endsWith(lower, ".lsm") || endsWith(lower, ".lif"))) {
        continue;
    }

    open(path);
    orig = getTitle();

    print("\\Clear");
    getDimensions(w, h, c, z, t);

    if (c < 5 || t > 1) {
        skipped++;
        closeAllImages();
        continue;
    }

    // ---------- split ----------
    run("Split Channels");

    ch4 = "C4-" + orig;
    ch5 = "C5-" + orig;

    if (!isOpen(ch4) || !isOpen(ch5)) {
        skipped++;
        closeAllImages();
        continue;
    }

    // ---------- JaCoP Manders ----------
    run("JACoP ", "imga=[" + ch4 + "] imgb=[" + ch5 + "] thra=" + thra + " thrb=" + thrb + " mm");

    logText = getInfo("log");

    m1_thresh = parseValueAfter(logText,
        "Manders' Coefficients (using threshold value of " + thra + " for imgA and " + thrb + " for imgB):\nM1=");
    m2_thresh = parseValueAfter(logText,
        "Manders' Coefficients (using threshold value of " + thra + " for imgA and " + thrb + " for imgB):\nM1=" + m1_thresh + " (fraction of A overlapping B)\nM2=");

    // ---------- write results ----------
    row = nResults;
    setResult("Image", row, orig);

    if (m1_thresh != "")
        setResult("M1_thresh", row, parseFloat(m1_thresh));
    else
        setResult("M1_thresh", row, NaN);

    if (m2_thresh != "")
        setResult("M2_thresh", row, parseFloat(m2_thresh));
    else
        setResult("M2_thresh", row, NaN);

    updateResults();

    processed++;
    closeAllImages();
}

setBatchMode(false);

// clear log and print completion
print("\\Clear");

saveAs("Results", inputDir + "jacop_combined_results.csv");

print("Done. Processed: " + processed + "  Skipped: " + skipped);
print("Saved: " + inputDir + "jacop_combined_results.csv");

// ---------- helpers ----------

function parseValueAfter(txt, key) {
    idx = indexOf(txt, key);
    if (idx < 0) return "";
    s = substring(txt, idx + lengthOf(key));

    end1 = indexOf(s, " ");
    end2 = indexOf(s, "\n");
    end3 = indexOf(s, "\r");

    end = -1;
    if (end1 >= 0) end = end1;
    if (end2 >= 0 && (end < 0 || end2 < end)) end = end2;
    if (end3 >= 0 && (end < 0 || end3 < end)) end = end3;

    if (end < 0) return s;
    return substring(s, 0, end);
}

function closeAllImages() {
    titles = getList("image.titles");
    for (ii = 0; ii < titles.length; ii++) {
        if (isOpen(titles[ii])) {
            selectWindow(titles[ii]);
            close();
        }
    }
}
```
