# Supplementary material for: Molecular and biophysical requirements for B cell receptor-dependent enhancement of dengue virus infection
Source: J Virol. 2026 Jul 20;100(8):e02216-25. doi: 10.1128/jvi.02216-25 (PMC13483438; doi:10.1128/jvi.02216-25)
Supplement: Supplementary file 2 [file jvi.02216-25-s0004.docx]

**Supplementary File 1.** Excel sheet with raw data used to create all figures and plots in this submission.

**Supplementary files 2.** Fiji macro code used for co-localization analysis shown in Figure 3.

**Video S1.** *3D renders of DENV internalization.* Gifs demonstrate individual cells framed in Figure 3 as representative of DENV binding or internalization in each timepoint. Scale bars: 5 μm. Cells were infected at 4˚C for 1 hr, then temperature shifted to 37˚C to allow for viral internalization. At the indicated timepoints, cells were fixed and permeabilized and stained with DAPI (blue), anti- DENV Env (yellow), and Rab7. GFP marks immortalized B cells. GIFs were made using Fiji from 38 Z-stacks. Data represents a single representative experiment of two performed.
