# Supplementary material for: Molecular and biophysical requirements for B cell receptor-dependent enhancement of dengue virus infection
Source: J Virol. 2026 Jul 20;100(8):e02216-25. doi: 10.1128/jvi.02216-25 (PMC13483438; doi:10.1128/jvi.02216-25)
Supplement: Supplementary file 1 [file jvi.02216-25-s0003.pdf]

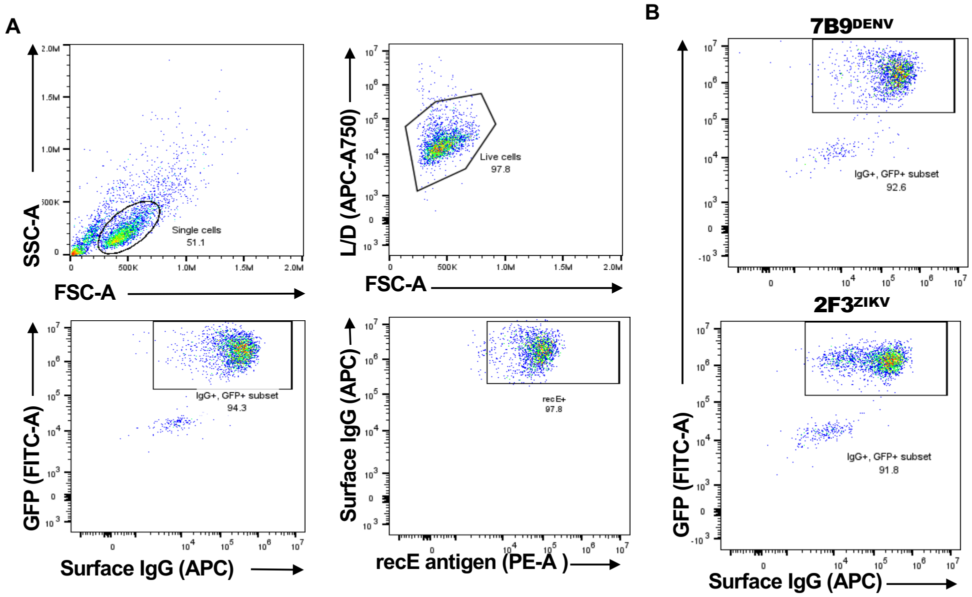

Fig. S1. *Flow Cytometry Gating of immortalized B cells and DENV recE dimer.* **(A)** Representative flow plots of gating utilized for DENV-2 and DENV-4 recE binding experiments in immortalized B cells. Immortalized cells are incubated with L/D APC-750 for 40min, washed, then incubated with 100ng of either recE-DV2 or recE-DV4 on ice for 30min. Followed by wash and final staining with anti-human IgG-APC for 30min. DENV recombinant envelope protein stabilized dimer (recE^{D}) is incubated with Streptavidin-PE for 30min before staining. (B) GFP and Surface IgG (BCR) is expressed in both sets 7B9^{DENV} and 2F3^{ZIKV-} cells.

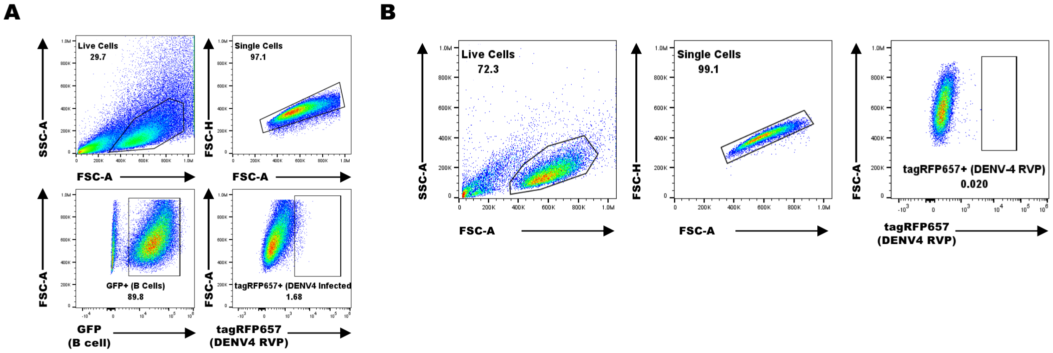

Fig. S2. *Flow Cytometry Gating of immortalized B cells and Raji DC-SIGN+ Cells*. **(A)** Representative flow plots of gating utilized for DENV-4 infection experiments in immortalized B cells. Immortalized B cells are co-cultured with CD40L expressing L cells, immortalized B cells are identified by GFP expression. DENV4 RVP Infection detected by expression of tagRFP657. **(B)** Representative flow plots of gating utilized for DENV-4 infection experiments in Raji DC-SIGN+ cells.

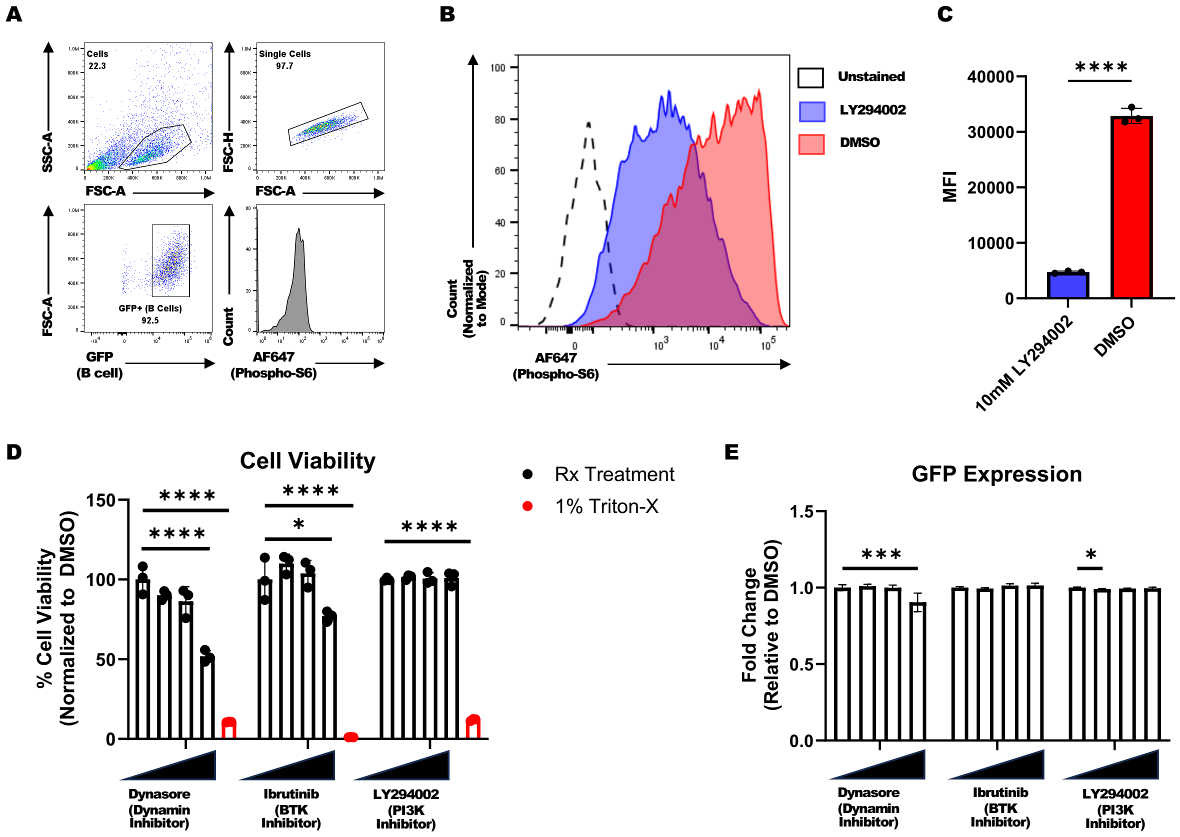

Fig. S3. *Characterization of pharmacologic inhibitors in immortalized B cells.* **(A)** Representative gating on immortalized B cells to detect PI3K activation quantified by expression of phosphorylated ribosomal protein S6. Detected using AF647 labelled anti-human phospho-S6 monoclonal cupk43k. **(B)** Representative histogram detecting phosphorylated S6 in immortalized human B cells treated with 10uM PI3K inhibitor LY294002 for 30 minutes. **(C)** Bar graph quantification of the mean fluorescence intensity of AF647 labelled anti-human phospho-S6 monoclonal cupk43k in DMSO (red) vs LY294002 treated (blue) cells. **** = p <0.0001, Unpaired T Test. **(D)** Bar graph quantification of cell viability by MTT assay in inhibitor treated cells. Cells treated for 4 hours with: DMSO, Dynasore (12.5uM, 50uM, 200uM), Ibrutinib (1.56uM, 12.5uM, 50uM), LY294002 (1.56uM, 12.5uM, 50uM), or 1% Triton (red). **(E)** Bar graph quantification of GFP expression in immortalized B cells following treatment with inhibitors described in D. Data collected 24 hours after treatment. Error bars +/- SEM. Experiments were conducted across single biological replicate in triplicate. Ordinary One-way ANOVA, * = p < 0.5, *** = p <0.001, **** = p <0.0001

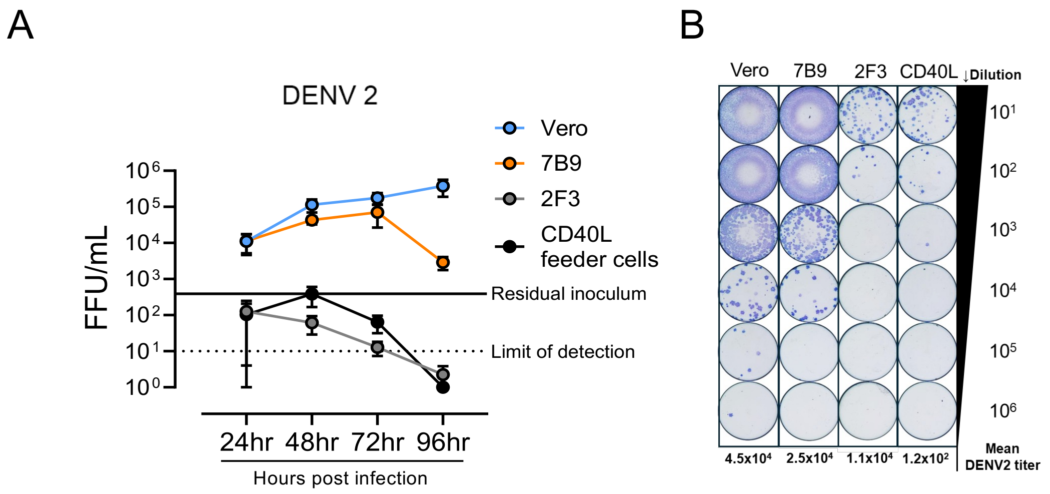

Fig S4. Generation of infectious DENV by DENV-specific B cells infected at high MOI. **A)** Vero cells, $7B9^{DENV}$ B cells, $2F3^{ZIKV}$ B cells, or CD40L-expressing fibroblast feeder cells were inoculated with DENV-2/NGC at MOI = 10 for 24 hrs at 37°C, washed, and cultured for the indicated times. Infectious virus in supernatants was determined by passage on Vero cells and assessed using a standard focus assay and data presented as focus forming units (FFU)/mL. **B)** Representative DENV-2 focus assay from supernatants collected 72 hrs post infection.
